# Toward the Discovery of Host-Defense Peptides in Plants

**DOI:** 10.3389/fimmu.2020.01825

**Published:** 2020-08-21

**Authors:** Benjamin Petre

**Affiliations:** Université de Lorraine, INRAE, IAM, Nancy, France

**Keywords:** antimicrobial peptides, peptide elicitors, concept transfer, plant immunity, defensins, thaumatin-like proteins

## Abstract

Defense peptides protect multicellular eukaryotes from infections. In biomedical sciences, a dominant conceptual framework refers to defense peptides as host-defense peptides (HDPs), which are bifunctional peptides with both direct antimicrobial and immunomodulatory activities. No HDP has been reported in plants so far, and the very concept of HDP has not been captured yet by the plant science community. Plant science thus lacks the conceptual framework that would coordinate research efforts aimed at discovering plant HDPs. In this perspective article, I used bibliometric and literature survey approaches to raise awareness about the HDP concept among plant scientists, and to encourage research efforts aimed at discovering plant HDPs. Such discovery would enrich our comprehension of the function and evolution of the plant immune system, and provide us with novel molecular tools to develop innovative strategies to control crop diseases.

## Introduction

**Figure 1:**
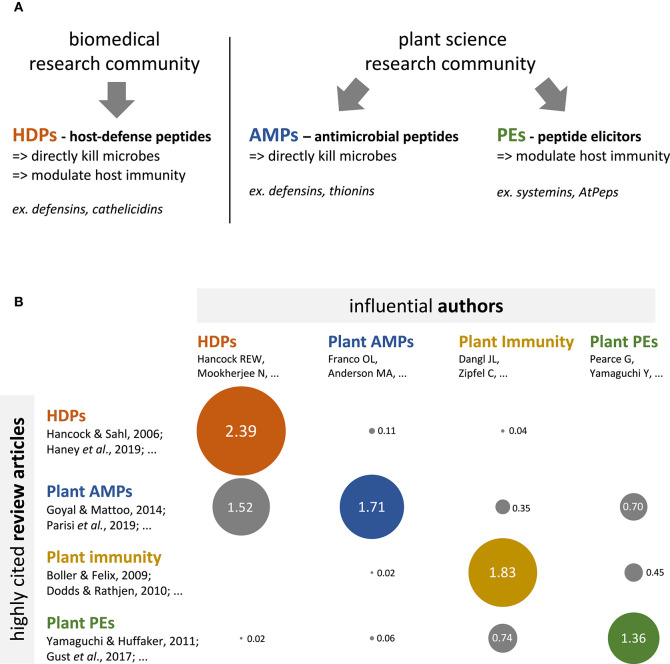
Biomedical and plant science fields have different conceptual frameworks regarding defense peptides, and limitedly cite each other's. **(A)** Conceptual frameworks pertaining to defense peptides in the biomedical and plant science research communities. The biomedical literature (left hand side panel) conceptualizes defense peptides as multifunctional molecules, with both antimicrobial and immunomodulatory activities, which are referred to as Host-Defense Peptides (HDPs). The plant science literature (right hand side panel) conceptualizes defense peptides as specialized molecules, which display either antimicrobial activities (antimicrobial peptides or AMPs) or immunomodulatory activities (peptide elicitors or PEs). **(B)** Bubble table chart depicting the results of a bibliometric analysis regarding the cross-referencing between HDP (red), plant AMP (blue), plant immunity (yellow), and plant PE (green) research communities. Numbers indicate the citation score for each field intersection (e.g., HDP vs. plant AMPs); that is expression of the average number of citations per author per article for a given field intersection (see Supplementary Methods for details). The diameter of the bubbles directly correlates with the indicated values. The analysis reveals that research communities are strongly compartmentalized, though some articles and 'transversal' authors evolve at the interface of the various research communities (see Supplementary Table 1 for details).

Defense peptides protect multicellular eukaryotes against pathogens such as microbes, and represent key tools to develop innovative disease control strategies in medicine and agriculture (1, 2). In biomedical sciences, defense peptides are often bifunctional, simultaneously able to directly kill microbes and to modulate host immunity. In 2006, Hancock and Sahl proposed to refer to these peptides as Host-Defense Peptides (HDPs) (Box 1) (3). The HDP concept has been rapidly captured by the biomedical research community, and has provided researchers with a robust conceptual framework to further discover and characterize HDPs (Figure 1A, left hand side) (4–6). In the plant science literature, no HDP (i.e., a peptide simultaneously able to kill pathogens and modulate host immune responses) has been convincingly reported so far (7, 8). The very concept of HDP is absent from the literature, and does not seem to have been captured by the research community.

Box 1 **Host-Defense Peptides - more than promiscuous AMPs.**

In this study, the term “Host-Defense Peptides” (HDPs) refers to defense peptides that exhibit two well-defined activities within the host immune system: an antimicrobial (or more broadly a biocidal) activity (i.e., direct killing of an invading organism) and an immunomodulatory activity (i.e., modulation of immune responses), as originally proposed (3). According to that definition, HDPs perform two functions that directly pertain to host immunity; and which have probably been selected throughout evolution. Understanding such HDPs is therefore key to better comprehend host immunity. Noteworthy, this article does not consider promiscuous plant AMPs (i.e., AMPs that display additional activities unrelated to the modulation of host immune responses) as HDPs.

Plant science literature currently categorizes defense peptides into two groups: antimicrobial peptides (AMPs) and peptide elicitors (PEs) (Figure 1A, right hand side). Plant AMPs are secreted proteins that interact with microbes and directly kill them (9, 10). Noteworthy, plant AMPs can be promiscuous and exhibit additional biological activities (8, 11–13), although the activities reported so far are unrelated to the modulation of immune responses; so that no plant AMP with immunomodulatory activity (i.e., an HDP) has been convincingly described to date. Plant PEs are small peptides that derive from larger precursor proteins and that function as ligands of cell-surface immune receptors to modulate plant immunity (14, 15). Thus, the current conceptual framework in plant science does not consider defense peptides as being able to simultaneously kill microbes and modulate plant immunity, therefore hindering efforts that could lead to the discovery of plant HDPs.

Conceptual frameworks guide research investigations and structure research communities. Indeed, I surmise that powerful concepts or models, often shared via influential review articles, shape the way researchers think and organize themselves. For example, the “zig-zag model” in plant immunity, proposed by Jones and Dangl in 2006, has cemented a robust research community and provided it with a strong conceptual framework to coordinate efforts and further investigate plant immunity (16). I claim here that the lack of awareness of the HDP concept within the plant science research community hinders the discovery of plant HDPs, as researchers lack the conceptual framework that would coordinate and encourage them to look for HDPs. Considering the innovative potential of HDPs, this ultimately deprives modern agriculture from the innovations it requires to be sustainable and efficient.

This perspective article has two goals: raise awareness of the HDP concept among plant scientists and encourage the search for HDPs in plants. To reach the first goal, I have used a bibliometric approach to identify articles and authors that may bridge biomedical and plant science communities and thus assist concept transfer. To reach the second goal, I have performed a literature survey to identify and list promising HDP candidates (i.e., AMPs with suspected immunomodulatory activities or PEs with suspected antimicrobial activities).

## A Bibliometric Analysis Reveals the Absence of the HDP CONCEPT in Plant Science, and Identifies Opportunities to Transfer the Concept From the Biomedical Field

To evaluate the status of the HDP concept in plant science, I performed a bibliometric analysis. I have first identified a set of 30 influential (i.e., highly cited) review articles published between 2009 and 2019, which focus on plant immunity, plant AMPs, or plant PEs (10 article for each category) (Supplementary Table 1). These 30 review articles have been collectively cited 6 813 times, and have probably shaped the dominant conceptual frameworks in their sub-fields. Secondly, I screened the main text of these articles for the term “HDP” or “host-defense peptides” using the Zotero key word search tool. I found zero occurrence of these terms. I further scrutinized the articles, and found no explicit reference to the HDP concept within them, although one article implicitly referred to the HDP concept (17). I conclude from that analysis that the most influential literature in plant science and plant immunity does not refer to the HDP concept, suggesting that plant science research community as a whole has not integrated this concept.

To identify influential articles and authors at the interface of the biomedical and the plant science communities (i.e., contact points) that could assist the HDP concept transfer, I have analyzed citation patterns between the plant science and the HDP literature. To this end, I have first identified a set of 10 influential (i.e., highly cited) review articles published between 2009 and 2019 that pertain to the HDP concept (Supplementary Table 1). These articles have been collectively cited 4 251 times, and are currently the most visible source of information about HDP in the academic literature. I have then identified the corresponding authors of all the review articles from Supplementary Table 1 (40 articles in total) and quantified how often they were cited in each of the review article, using a citation score that disregarded self-citations (Figure 1B; see Supplementary Methods for details). The analysis first shows strong 'intra-community' citation, as the articles from one particular sub-field (HDP for instance) cite predominantly the authors from the same community (average citation score of 1.83). In contrast, “cross-community” citation (i.e., citation between different research communities) is lower (average citation score of 0.33), with even null values at the intersection of HDP articles *vs*. plant PEs authors and plant immunity articles vs. HDP authors; this altogether suggests no—or seldom—cross-community information flow. Finally, and most interestingly, the analysis shows that the plant AMP article set cites authors from the three other communities (average citation score of 0.86). A further detailed investigation of the citation pattern revealed a handful of specific articles and authors that cite—and are cited—beyond the communities boundaries (Supplementary Table 1) (10, 17–21). Such “transversal” articles and authors are probably and simultaneously knowledgeable about the HDP concept and visible within the plant science research community; they are therefore in a good position to assist cross-community concept transfer.

## Candidate HDPS in Plants: Defensins, Thaumatin-like Proteins, and Others

To encourage investigations aimed at discovering HDPs in plants, I identified and listed what are in my view the most promising candidate HDPs. To do so, I screened the literature for reports of AMPs that exhibit an additional activity that could be related to modulation of the plant immune system, or for PEs (or their precursors) that might exhibit antimicrobial activities. In total, I found six such peptides; two that belong to the defensin superfamily (alfalfa MsDef1 and tomato DEF2), two that belong to—or derive from—the thaumatin-like protein (TLP) superfamily (sweet potato IbACP and european plum PdPR5-1), and two that do not belong to large conserved multigene families (poplar RISP and pepper CaAMP1) (Table 1).

**Table 1:** Plant HDP candidates.

**Peptide name**	**Peptide information**	**Peptide origin**	**Antimicrobial activity**	**Immunomodulation-related activity**	**References**
MsDef1	MsDef1 (*Medicago sativa* Defensin 1) belongs to the defensin family	Alfalfa (*Medicago sativa*)	Purified MsDef1 inhibits fungal growth	Purified MsDef1 inhibits root growth (suggesting immune response activation)	(22, 23)
DEF2	DEF2 belongs to the defensin family	Tomato (*Solanum lycopersicum*)	DEF2-containing foliar extracts inhibit fungal growth	Ectopically-expressed DEF2 perturbs tomato plant development and reduces seed production (trade-off between growth and defense)	(24)
IbACP	IbACP (*Ipomea batatas* anti-cancer peptide) probably derives from the N-terminus of a PR-5/TLP precursor	Sweet potato (*Ipomea batatas*)	IbACP precursor belongs to the TLP family, whose members are well-established AMPs	Purified IbACP induces tomato cell culture alkalinisation (suggesting immune response activation)	(25)
PdPR5-1	PdPR5-1 (*Prunus domestica* Pathogenesis-related Protein 5 1) belongs to the Thaumatin-Like Protein (TLP) family	European plum (*Prunus domestica*)	PdPR5-1 belongs to the TLP family, whose members are well-established AMPs	Ectopically-expressed PdPR5-1 induces the expression of plant defense pathways (suggesting immune response activation)	(26)
CaAMP1	CaAMP1 (*Capsicum annuum* antimicrobial protein 1) is a 186-amino-acid antimicrobial protein from pepper	Pepper (*Capsicum annuum*)	Purified CaAMP1 inhibits the growth of bacteria, fungi, and oomycetes	Ectopically-expressed CaAMP1 modulates the expression of plant defense-related proteins (suggesting immune response modulation)	(27, 28)
RISP	RISP (Rust-Induced Secreted Protein) is a 82-amino-acid peptide that belongs to a Salicaceae-specific family of Cysteine-rich, cationic, secreted peptides	Poplar (*Populus trichocarpa*)	Purified RISP inhibits fungal growth	Purified RISP triggers poplar cell culture alkalinisation (suggestive of immune response activation)	(7)

Among the six peptides listed in Table 1, four (MsDef1, DEF2, CaAMP1, and RISP) were reported as AMPs that directly inhibit microbe growth (7, 22, 24, 27). In contrast, two peptides (IbACP and PdPR5-1) have been shown to function as PEs, and their direct antimicrobial activity was not tested (25, 26). However, both PdPR5-1 and the probable precursor of IbACP are members of the TLP superfamily, which is a well-characterized AMPs family in plants (29). I therefore consider likely that PdPR5-1 and IbACP precursor both display a direct antimicrobial activity.

The six above-mentioned peptides alter plant physiology in a way that suggests a potential role as immunomodulators. On the one hand, purified IbACP and RISP both trigger rapid plant cell culture alkalinization, while purified MsDef1 inhibits plant root growth (7, 23, 25). Both cell culture alkalinization and root growth inhibition are hallmarks of—and are commonly used as a readout for—the activation of plant immunity (30, 31). This suggests that these three peptides can directly control immunity, potentially working as a ligand to an immune receptor, as hypothesized for RISP (32). On the other hand, ectopically-expressed DEF2, PdPR5-1, and CaAMP1 affect plant physiology. PdPR5-1 and CaAMP1 modulate the expression of defense-related genes, or genes that participate in defense pathways, suggesting that the expression of the peptides in the plant stimulates immune responses (26, 28). DEF2 alters plant growth, and notably reduces seed production (24). Considering the trade-off that exists between growth and defense in plants (33), this alteration could result from an alteration of the growth/defense balance.

Altogether, this set of observations suggests that the six above-mentioned peptides represent priority candidates in the search for plant HDPs. It also indicates that HDP candidates can be detected in well-characterized and conserved plant AMP gene families (such as TLPs or defensins), which would facilitate further functional investigations.

## Discussion

In this perspective article, I have combined a bibliometric analysis with a literature survey to evaluate the status of the HDP concept in plant science and to encourage research efforts aimed at discovering plant HDPs. The bibliometric analyses showed that the HDP concept has not been captured by the plant science community, but also revealed interfacial research communities that could assist concept transfer. The literature survey identified a list of six defense peptides that I propose to consider as priority HDP candidates.

To bolster the effort aimed at discovering HDPs in plants, I see two obvious perspectives. Firstly, we could test known AMPs for additional immunomodulatory activities. In most cases, this task would take advantage of available purified peptides (usually used to demonstrate the antimicrobial activity) that could be directly used in assays that rely on exogenous peptide application (e.g., cell culture alkalinization or root-growth inhibition assays). Secondly, we could test known PEs, as well as their precursor proteins, for direct antimicrobial activities. Priority could be given to PE precursors that are predicted to be secreted out of the cells (e.g., Hydroxyproline-rich systemins; HypSys) and/or that are organized into well-characterized multigene families with known immunomodulatory roles (e.g., Rapid Alkalinisation Factors; RALF). Completing such tasks may rapidly help us determine whether defense peptide with HDP-like properties evolved in plants. An alternative to these two approaches consist in searching for synthetic peptides with HDP-like properties (34–36). Although such an approach would not inform much about the evolution and the function of the plant immune system, it would still provide us with valuable tools to develop phytosanitary products, such as peptide-based biopesticides for instance, to protect crops from diseases.

## Data Availability Statement

All datasets presented in this study are included in the article/Supplementary Material.

## Author Contributions

BP is the only author of the manuscript, he prepared the manuscript himself.

## Conflict of Interest

The author declares that the research was conducted in the absence of any commercial or financial relationships that could be construed as a potential conflict of interest.
