# Supplementary material for: Toward the Discovery of Host-Defense Peptides in Plants
Source: Front Immunol. 2020 Aug 21;11:1825. doi: 10.3389/fimmu.2020.01825 (PMC7472956; doi:10.3389/fimmu.2020.01825)
Supplement: Supplementary file 2 [file Data_Sheet_1.docx]

Supplementary Methods

# Building of the literature corpus

To build a corpus of 40 influential review articles published between 2009 and 2019 in the fields dealing with host-defense peptides (HDPs), plant antimicrobial peptides (AMPs), plant immunity, and plant peptide elicitors (PEs), I used the 'basic search' tool of Web of Science website to perform key word searches (with the above-mentioned terms for each field), and filtered the results to display only 'review articles' by sections of three to four years (2009-2012; 2013-2016; 2017-2019, to crudely correct number of citation bias due to publication date). I then sorted the results by citation (most to least), and I scrutinized the output of the search to eliminate out-of-scope articles and articles that did not significantly share conceptual insights, to finally select 10 articles in each of the four categories above-mentioned. I then downloaded the 40 .pdf files of the review articles and incorporated them into four dedicated sub-collections (one for each field abovementioned) in the Zotero software version 5.0.85 (<https://www.zotero.org/>).

# Keyword searches and quantification.

To identify the review articles containing a given keyword (ex. 'HDP') or author name (ex. Hancock), I used the search tool integrated into Zotero, with the 'everything' parameter. Then, in order to accurately quantify the occurrence of the keyword or author name in the articles, I manually inspected the manuscript content to count the correct keyword or author name occurrence using the .pdf reader Preview version 10.1. I recorded the data gained through that screening into a spreadsheet. Notably, for the citation pattern analysis, I recorded the number of times an author name appeared in the reference section of an article, using an 'article x author' matrix (Supplementary Table S1). Within the matrix, I indicated the numbers corresponding to self-citation (the author is cited within an article he or she cosigned) between brackets, and did not consider them for further analysis.

# Computation of the citation score and design of the bubble table chart.

To calculate the citation score for each field intersection (*e.g.* HDP vs. plant AMPs), I calculated the average of the values displayed in the corresponding matrix area in Supplementary Table S1, which I then divided by the total number of cells in the area (*i.e.* the citation score can then be interpreted as the expression of the average number of citations per author per article for a given field intersection). I then used all the 16 citation scores (16 field intersections compose the matrix) to build a bubble table chart with the drawing tools of Microsoft PowerPoint software, using the citation score values as the diameter of the bubbles, so that the diameter of the bubbles positively correlates with citation score.
